# The Effect of Acupressure on Fasting Blood Glucose and Glycosylated Hemoglobin Levels in Diabetic Patients: A Randomized Controlled Trial

**DOI:** 10.30476/ijcbnm.2021.86059.1318

**Published:** 2021-04

**Authors:** Sied Saeed Najafi, Hassan Ghorbani, Amin Kordi Yoosefinejad, Majid Najafi Kalyani

**Affiliations:** 1 Department of Medical Surgical Nursing, School of Nursing and Midwifery, Shiraz University of Medical Sciences, Shiraz, Iran; 2 Student Research Committee, School of Nursing and Midwifery, Shiraz University of Medical Sciences, Shiraz, Iran; 3 Department of Physical Therapy, School of Rehabilitation Sciences, Shiraz University of Medical Sciences, Shiraz, Iran; 4 Rehabilitation Sciences Research Center, Shiraz University of Medical Sciences, Shiraz, Iran

**Keywords:** Acupressure, Glycosylated hemoglobin, Randomized controlled trial

## Abstract

**Background:**

Diabetes is the most common endocrine disorder. Non-pharmacological methods can be used for treatment of these patients. The present study aimed to investigate the effect of acupressure point on fasting blood glucose and glycosylated levels of diabetic patients.

**Methods:**

This clinical trial was conducted on 102 patients who referred to Motahari Clinic of Shiraz during May-June in 2018. The participants were selected based on simple random sampling and divided into three groups via permuted block randomization. The control group only received the pharmacological treatments. The intervention group received acupressure at ST36 point in addition to medications. The placebo group also received medications and acupressure at a fake point. The intervention was carried out for six minutes (three minutes for each lower extremity), three sessions a week for 12 weeks. Fasting blood glucose and glycosylated hemoglobin levels were checked in all patients immediately after the intervention. The data were analyzed using Chi-square, paired t-test, and ANOVA by the SPSS statistical software, version 21, and P<0.05 was considered statistically significant.

**Results:**

The results showed no significant differences among the three groups’ blood glucose mean levels before (P=0.89) and after the intervention (P=0.36). However, a significant difference was observed in the intervention group’s glycosylated hemoglobin mean levels before (8.61±1.96) and after the intervention (8.1±1.62) (P=0.02).

**Conclusion:**

In sum, the study indicated that acupressure could only be effective in reducing the glycosylated hemoglobin in the intervention group. Thus, further larger studies are recommended to evaluate the effectiveness of this technique.

**Trial Registration Number:** IRCT20111224008505N47.

## INTRODUCTION

Diabetes mellitus is one of the most common chronic disorders around the world. It is the third cause of mortality in the world. ^1 , 2^ In 2015, the International Diabetes Federation (IDF) reported that 415 million adults suffered from diabetes worldwide and this figure was expected to reach 642 million by 2040. ^3^ As a report in 2018, there are more than 500 million cases of type 2 diabetes mellitus (T2DM) worldwide. ^4^ T2DM is a chronic progressive disorder, which is identified by insulin secretion disorder and insulin resistance in the liver, adipose tissue, and skeletal muscles. ^5 , 6^

Measurement of glycosylated hemoglobin level is one of the accurate standard methods for long-term management of diabetes, which indicates the mean blood glucose concentration in a 2-3 month period. ^7^ Evidence has demonstrated that reduction of glycosylated hemoglobin level by some percentage could decrease the risk of diabetes complications to a considerable extent. ^8^

Generally, management and treatment of diabetes involves a wide spectrum of pharmacological and non-pharmacological methods. ^9^ Non-pharmacological methods include the diet, exercise, and consultation with respect to Complementary and Alternative Medicine (CAM) including acupuncture, massage therapy, biofeedback, yoga, and herbal medications, which play a critical role in reduction of blood glucose level. ^10^ CAM is a non-pharmacological method, which has been recently applied in management and treatment of diabetes. CAM contains a wide variety of interventions, measures, and exercises for prevention and treatment of diseases and improvement of health. ^11^ One of CAM techniques is acupressure in which pressure is exerted on the body reflex points. ^12^ One of such reflex points is acupressure point (ST36), which is associated with the pancreas and other internal organs. This point is located four finger widths down the patella. ^12^

As mentioned above, in comparison to fasting blood glucose, glycosylated hemoglobin is a more accurate indicator of blood glucose level in the long run. However, previous studies used different times, ^12^ points, ^13^ and outcomes and have not assessed the glycosylated hemoglobin levels. ^12^ Regarding the existing gap and importance of using a simple and easy intervention, the present study aimed to investigate the effect of acupressure at ST36 point on the main outcomes (fasting blood glucose and glycosylated hemoglobin levels) in patients with T2DM. 

## MATERIALS AND METHODS

This randomized controlled clinical trial (IRCT20111224008505N47) was conducted on patients with T2DM referred to the diabetes clinic of Motahari polyclinic in Shiraz for follow-up during May-June 2018. 

In this study, based on the research performed by Yodsirajinda, ^14^ considering μ_1_=9.6, μ_2_=8.6, σ_1_=1.37, σ_1_=0.96, α=0.05, and β=0.1 (power=1-β=90%), the sample size was estimated as 31 participants in each study group. Considering the 10% probability of loss, this increased to 34 participants in each group.

$n=\frac{{\left(Z_{\mathrm{1-\alpha /2}}+Z_{\mathrm{1-\beta}}\right)}^{2}\times (\sigma_{1}^{2}+\sigma_{2}^{2})}{{\left(\mu_{1}-\mu_{2}\right)}^{2}}$

$n=\frac{{\left(1.96+1.281\right)}^{2}\times (1.37^{2}+0.96^{2})}{{\left(9.6-8.6\right)}^{2}}$

**Figure 1:**
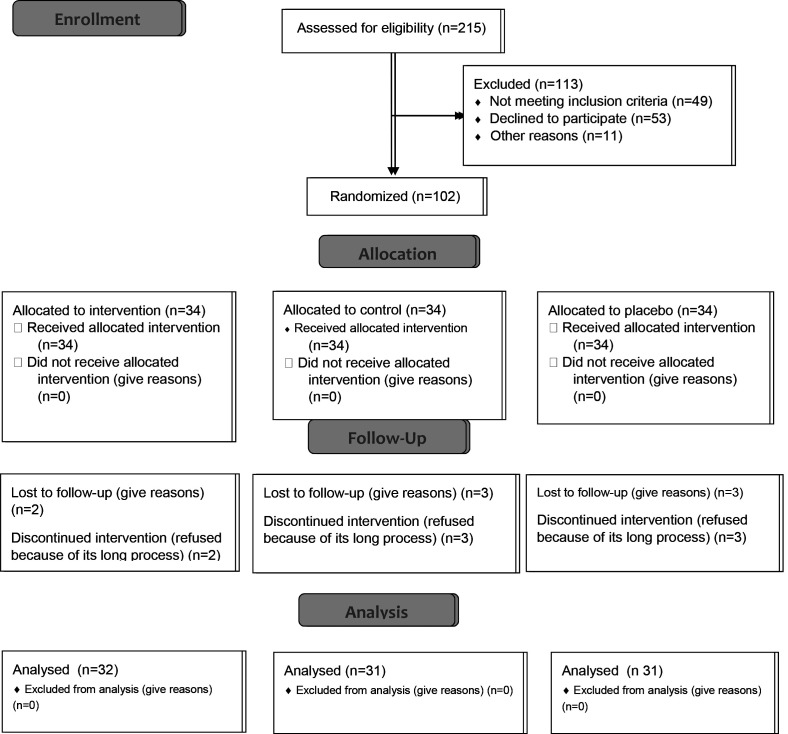
Flow diagram of patients’ progress through the stages of randomized controlled trial

The inclusion criteria of the study were age >18 years, diagnosis of T2DM by an internal specialist, and affliction with diabetes for at least three years. The exclusion criteria were suffering from peripheral vascular disease (diagnosed by a physician), inflammation, skin ulcer, fracture, musculoskeletal disorder at ST36 zone, cancer, and allergy to massage. The patients who were absent for more than one session were also excluded from the study (Figure 1). 

This study was approved by the Ethics Committee of Shiraz University of Medical Sciences, Shiraz, Iran (IR.SUMS.REC.1396.183). The study participants were entered into the study after signing written informed consent forms. The patients were also ensured that their information would be kept confidential and they could withdraw from the study whenever they desired.

The data were collected using a demographic information form. Blood samples were also taken from the participants to measure their fasting blood glucose and glycosylated hemoglobin levels. To measure glycosylated hemoglobin and fasting blood sugar, the laboratory expert took 2cc of blood from patients under fasting conditions and then measured it with a Pars kit, which is a standard kit and product of Pars Azmoun Company. Reliability of this kits was measured 3 times, revealing 99% correlation. The participants were divided into the intervention, placebo, and control groups via permuted block randomization (ABC) using a random number generator. This study was a double-blind trial. All participants and outcome evaluators were blind. Considering homogenization, all patients and their companions received face-to-face training and educational pamphlets regarding physical activity and adherence to eating diet.

The control group only received the pharmacological treatments prescribed by the physician. After acquiring the necessary skills in the field of acupressure under supervision of a physiotherapist, the second author started sampling. The intervention group received acupressure at ST36 point (four finger widths down from the patella) in addition to medications. The placebo group also received medications and acupressure at a fake point (1.5 cm lower than the intended point). The acupressure procedure in this study consisted of one press per second, and, after five continuous one-second presses and a two-second rest, the cycle was repeated for three minutes. In doing so, 3-5 kg/cm2 pressure was exerted on the intended points at the depth of 1.5 cm by using a specific equipment under the supervision of an expert. The intervention was carried out for six minutes (three minutes for each lower extremity) three sessions a week for 12 weeks. ^12 , 15^ The reason for choosing the time of 12 weeks was because glycosylated hemoglobin shows the levels of blood sugar during the last 2 to 3 months; to observe the necessary changes in this factor, we had to prolong the intervention for at least 12 weeks. The reason for choosing the time of 6 minutes was because acupressure was constantly tedious for patients, and by assessing several sources, this time was chosen that was not boring for the patients.

In the first week, acupressure was done for the patients individually. In so doing, the intervention was done by the second author and his assistant during the three sessions of the first week. The patients or their companions were also trained about performing acupressure, finding acupressure point, and exerting pressure at this point. Afterwards, the patients or their companions were responsible for continuing the intervention. It should be noted that the researcher made phone contacts with the patients twice a week during the second and third weeks and once a week within the last 9 weeks to ensure that the intervention was done by the patients. 

Fasting blood glucose and glycosylated hemoglobin levels were checked in all patients at the end of 12 weeks of the intervention. At the end of the study and after data analysis, the precise method of exerting pressure at the acupressure points was taught to the patients in the placebo and control groups. It should be mentioned that eight patients refused to continue the study because they were reluctant to continue the intervention due to its long process.

Mean and Standard Deviation (SD) were used to compute descriptive statistics. The data were analyzed using Chi-square, paired t-test, and ANOVA. All analyses were performed using the SPSS statistical software under the license of IBM, version 21, and P<0.05 was considered statistically significant.

## RESULTS

The results revealed no significant differences among the three groups with respect to demographic variables of the participants. This indicated that the three groups were matched regarding the study variables (Table 1). Comparison of three groups in term of age (P=0.79) and duration of suffering from diabetes (P=0.82) showed that there was no statistically significant difference. Kolmogorov–Smirnov test was used to check the normality of the data.

**Table 1:** Distribution of the participants’ demographic variables in the three groups

Variable		Intervention group (n=32) N (%)	Control group (n=31) N (%)	Placebo group (n=31) N (%)	P value*
Sex	Male	6 (18.8)	9 (29.0)	8 (25.8)	0.62
	Female	26 (81.2)	22 (71.0)	23 (74.2)	
Education level	Illiterate	0 (0.0)	10 (32.3)	4 (12.9)	0.30
	Primary school	13 (40.5)	12 (38.7)	11 (35.5)	
	Middle school	6 (18.8)	1 (3.2)	4 (12.9)	
	Diploma	7 (21.9)	6 (19.4)	9 (29.0)	
	4Academic	6 (18.8)	2 (6.4)	3 (9.7)	
Use of oral diabetes medications	Yes	28 (87.5)	26 (83.9)	27 (87.1)	0.93
	No	4 (12.5)	5 (16.1)	4 (12.9)	
Insulin therapy	Yes	10 (31.3)	11 (35.5)	9 (29.0)	0.88
	No	22 (68.7)	20 (64.5)	22 (71.0)	
Use of CAMa	Yes	9 (28.1)	9 (29.0)	6 (19.4)	0.60
	No	23 (71.9)	22 (71.0)	25 (80.6)	
Exercise activities	Yes	24 (75.0)	23 (74.2)	16 (51.6)	0.09
	No	8 (25.0)	8 (25.8)	15 (48.4)	
Tobacco use	Yes	0 (0.0)	4 (12.9)	6 (19.4)	0.26
	No	32 (100.0)	27 (87.1)	25 (80.6)	

* Chi-square; aComplementary and Alternative Medicine

The results showed no significant differences among the three groups’ blood glucose mean levels before (P=0.89) and after the intervention (P=0.36). The results also indicated no significant differences among the three groups with respect to glycosylated hemoglobin mean levels before (P=0.37) and after the intervention (P=0.60) (Table 2).

**Table 2:** Mean levels of blood glucose and glycosylated hemoglobin between and within the three groups

Variable		Before (Mean±SD)	After (Mean±SD)	P value*
Blood glucose level	Intervention group	162.75±61.09	143.34±49.75	0.07
	Placebo group	154.58±75.51	146.45±60.81	0.41
	Control group	160.1±76.66	165.0±80.96	0.54
P-value**		0.89	0.36	
Glycosylated hemoglobin level	Intervention group	8.61±1.96	8.1±1.62	0.02
	Placebo group	8.07±1.52	7.99±1.59	0.73
	Control group	8.06±1.77	8.39±1.7	0.12
P value**		0.37	0.60	

* Paired t-test,

** One-way ANOVA

The results revealed no significant differences in the glycosylated hemoglobin mean levels in the control (P=0.12) and placebo (P=0.73) groups before and after the intervention. However, a significant difference was observed in the intervention group in this regard before and after the intervention (P=0.02) (Table 2). The results also revealed no significant differences in the blood glucose mean levels in the control (P=0.54), placebo (P=0.41), and intervention groups (P=0.07) before and after the intervention. (Table 2).

## DISCUSSION

The results of the present study revealed no statistically significant differences in blood glucose levels within the groups and among groups before and after the intervention. There was no statistically significant difference in the glycosylated hemoglobin levels among the groups before and after the intervention, but within the groups there was a statistically significant decrease only in the intervention group after the intervention. 

In the same line, a study compared four groups (control, Electroacupuncture (EA)-5 Hz, EA-50 Hz, and EA-100 Hz) and reported no significant differences among the groups regarding fasting blood glucose level. ^16^ Considering the glycosylated hemoglobin level, a significant difference was observed only in the EA-5 Hz group. It should be noted that electroacupuncture was used in the mentioned study, ^16^ while acupressure was employed in the present research. Indeed, four acupoints, namely Pishu (BL20), Shenshu (BL23), acupressure point (ST36), and Sanyinjiao (SP6), were used in the former study, while only acupressure point (ST36) was used in the present one.

The results of another study showed a significant decline in the fasting blood glucose level in the intervention group compared to the control group, ^13^ which is inconsistent with the results of the current investigation. The discrepancy between the results might be due to the fact that only one acupressure point was used in the present study, while acupressure points as well as Liver-3 (LIV-3), Kidney-3 (KID-3), and Spleen-6 (SP-6) acupoints were used in the other one. 

A study demonstrated a significant decrease in the intervention group’s blood glucose mean level in the post-test compared to the baseline. ^17^ However, no significant changes were detected in the placebo group. These results were not in agreement with those of the current study, which might be due to the difference in the duration of the interventions. In the mentioned research, the intervention was performed in ten 60-90 minute sessions. ^17^ In the present study, on the other hand, the intervention was carried out for six minutes three sessions a week for 12 weeks. Moreover, only acupressure at one acupressure point was applied in this study. However, previous researchers made use of acupressure as well as hypnotherapy and meditation, which might have resulted in the decline in blood glucose level. ^17^

A study showed that acupressure at ST36 acupoint was effective in reducing the blood glucose level in the intervention group compared to the control group, ^12^ which is not in the same line with the results of the present study. Blood glucose level decreased by nearly 240 units in that study, ^12^ but only by 21 units in the current research. The discrepancy between the results might result from the difference in the duration of performance of acupressure. This technique was applied for 30 minutes in every treatment session in the previous study, ^12^ but for six minutes in the present one.

Another study revealed a significant decline in the intervention group’s glycosylated hemoglobin level in comparison to the control group when acupressure was done at sp-6 point for three minutes three sessions a week for 12 weeks, ^18^ which is consistent with the results of the current study in the intervention group. However, the intervention was done for six minutes three sessions a week for 12 weeks in the current study. The same results show that applying acupressure is effective in reducing the glycosylated hemoglobin level as the duration and the point were different.

Acupressure at the acupressure point (ST36) stimulates the hypothalamic-pituitary actuator that activates the neurotransmitters, thereby regulating the endocrine glands. ^12^ The impact of induction of alternative and complementary therapies, such as acupuncture and acupressure, is well known. ^19^ Therefore, based on the results of this study and the above-mentioned studies and considering the effectiveness of acupressure at acupressure point (ST36) in reducing the glycosylated hemoglobin levels only in the intervention group in the present study, it can be concluded that the difference among the results might be due to utilization of different acupressure points, various intervention periods, and use of different alternative and complementary methods.

One of the most strengths of this study was applying acupressure on a specific point as a new acupressure approach for diabetic patients. Furthermore, evaluating the glycosylated hemoglobin along with blood sugar as a more precise index was another strength of this study. One of the potential limitations of this study, compared to other studies, may be the low duration of acupressure because it was constantly tedious for patients. Another limitation of the present study was that the intervention was performed by the patients for 11 weeks.

## CONCLUSION

Generally, the study results indicated that acupressure could only be effective in reducing the glycosylated hemoglobin in the intervention group. As a significant difference was shown in the intervention group’s glycosylated hemoglobin mean levels before and after the intervention, it can be used by nurses as an inexpensive and easy approach for management of the outcomes of diabetic patients. Therefore, further studies with long-term duration of acupressure and in different acupressure points are recommended to be conducted in diabetic patients. 
